# ﻿Three new species of the genus *Clavulina* (Hydnaceae, Cantharellales) from North China based on morphological and phylogenetic analysis

**DOI:** 10.3897/mycokeys.108.124004

**Published:** 2024-08-22

**Authors:** Yue Gao, Xin Tong, Hao Zhou, Hai-Qi Wang, Cheng Li, Cheng-Lin Hou

**Affiliations:** 1 College of Life Science, Capital Normal University, Haidian, 100048, Beijing, China Capital Normal University Beijing China; 2 Department of Life Sciences, National Natural History Museum of China, Tianqiaonandajie 126, Dongcheng, 100050, Beijing, China Department of Life Sciences, National Natural History Museum of China Beijing China

**Keywords:** Clavarioid fungi, systematics, taxonomy, three new taxa

## Abstract

*Clavulina* possesses important ecological and economic value and has attracted extensive attention from mycologists. Macrofungal diversity is high in China, but *Clavulina* species have not been thoroughly studied. In this study, based on morphological evidence and phylogenetic analyses of the nucleotide sequences of three loci (nrITS, nrLSU, and *rpb2*), three new species of *Clavulina* from North China were identified. Morphologically, *Clavulinachengdeensis* is characterized by its white to dirty white basidiomata with somewhat pale orange tips and somewhat wrinkled hymenium. *Clavulinagriseoviolacea* is characterized by its gray to dark grayish violet basidiomata, with a sometimes-white stipe base, monopodial or irregularly polychotomous toward branch apices. *Clavulinapallida* is characterized by its white to pale cream white basidiomata with somewhat orange tips. Phylogenetically, the three new species form three independent branches with high support values in the phylogenetic tree.

## ﻿Introduction

*Clavulina* J. Schröt. (Hydnaceae, Cantharellales), with *Clavulinacristata* (Holmsk.) J. Schröt. as a type species, was established in 1888 (Schröter 1888; He et al. 2019). *Clavulina* is a clavarioid fungi, characterized by clavarioid to coralloid, simple or branched basidiomata with amphigenous hymenia, cylindrical to subclavate basidia with two or more cornuted sterigmata, often with postpartal septa after the release of the basidiospores, and smooth, globose to subglobose basidiospores (Corner 1950, 1970; Petersen 1988a; Thacker and Henkel 2004; Henkel et al. 2005; Olariaga et al. 2009; Henkel et al. 2011; Uehling et al. 2012a). Most *Clavulina* species are ectomycorrhizal fungi associated with diverse trees (Tedersoo et al. 2003; Tedersoo et al. 2010; Smith et al. 2011; Wazny 2014). Some species are edible or have medicinal value (Dai and Yang 2008; Dai et al. 2010).

The delimitation of *Clavulina* species is a challenge because they appear to be “simple” in morphology (Donk 1933, 1964; Corner 1950, 1970; Petersen 1988a, 1988b). Molecular phylogenetic analysis based on DNA sequences has improved the identification of species, especially those that cannot be distinguished by morphology alone (Hibbett et al. 1997; Pine and Donoghue 1999; Larsson et al. 2004; Thacker and Henkel 2004; Binder et al. 2005; Moncalvo et al. 2006). Currently, approximately 99 species of *Clavulina* have been described from temperate and tropical forests worldwide, with nearly half of these species found in the tropics (Wu et al. 2019; Yuan et al. 2020; de Meiras-Ottoni and Gibertoni 2023; Huang et al. 2023; Salas-Lizana et al. 2023). In China, 11 *Clavulina* species have been reported on the basis of morphological and molecular analyses, most of which are found in subtropical regions, viz. *C.bessonii* (Pat.) Corner, *C.castaneipes* (G.F. Atk.) Corner (= *C.ornatipes* (Peck) Corner), *C.coralloides* (= *C.cristata*), *C.cinerea*, *C.rugosa* (Teng 1963; Zang et al. 1996; Li et al. 2015), *C.livida* (He et al. 2016), *C.flava*, *C.purpurascens* P. Zhang (Wu et al. 2019), *C.baiyunensis* X.X. Huang & L.H. Qiu, *C.minor* X.X. Huang & L.H. Qiu, and *C.lilaceorosea* X.X. Huang & L.H. Qiu (Huang et al. 2023).

Recently, a number of *Clavulina*-like samples were collected during an investigation of the Yanshan Mountains in North China, a warm temperate region. Three new species were recognized on the basis of morphological and molecular data. In this paper these new species are described and illustrated. The nuclear ribosomal internal transcribed spacer (nrITS), the large subunit of the nuclear ribosomal RNA (nrLSU), and the RNA polymerase II second largest subunit (*rpb2*) were sequenced from dried basidiomata.

## ﻿Materials and methods

### ﻿Collecting and site description

The specimens were collected from 2017 to 2023 in Beijing, Hebei Province, and Tianjin, North China. These areas have a warm temperate continental monsoon climate and a diverse assortment of plants. The dominant forest types are deciduous broad-leaved forest and mixed coniferous and broad-leaved forest. The dominant trees include *Quercusmongolica* Fisch. ex Ledeb., *Betulaplatyphylla* Suk., *Abiesnephrolepis* (Trautv.) Maxim., *Populustomentosa* Carrière, and *Pinustabuliformis* Carr. (Wang et al. 2021; Zhou et al. 2022). The annual precipitation is approximately 700 mm. The altitude ranges from 200 to 2200 m. The collected specimens were dehydrated using an electric dryer (Dorrex) at 50 °C and kept in the Herbarium of the College of Life Science, Capital Normal University, Beijing, China (BJTC).

### ﻿Morphological observation

Macroscopic characteristics were documented from dried specimens and photographs, while thin sections of specimens mounted in 3% potassium hydroxide (KOH) or sterilized water were analyzed for microscopic features. The morphology and dimensions of their microscopic structures were observed and recorded using a light microscope [Olympus DP71, Tokyo, Japan]. In the description of basidiospores, the abbreviation *n*/*m*/*p* means that *n* basidiospores were measured from *m* basidomata of *p* collections. The measurements and *Q* values were presented in the form of (a)b–c (d), in which “b-c” contained a minimum of 90% of the measured values, and extreme values (a and d) were given in parentheses. *Q* represents the ratio of the length to width of basidiospores, and *Q_m_* represents the average *Q* value of all basidiospores measured ± the sample standard deviation (Huang et al. 2023). The nomenclatural details were submitted to MycoBank. Color designation was referred to the website colorhexa (https://www.colorhexa.com).

### ﻿DNA extraction, PCR amplification and sequencing

DNA was extracted with an M5 Plant Genomic DNA Kit [Mei5 Biotechnology, Co., Ltd., China]. The obtained DNA was dissolved in 1 × TE buffer and stored at –20 °C for later use. PCRs were performed in a Bio-Rad S1000TM Thermal Cycler [Bio-Rad Laboratories, Inc., USA]. The primer set ITS1f/ITS4 (White et al. 1990) was used to amplify the nrITS region. The primer set LR5/LR0R (Vilgalys and Hester 1990) was used to amplify the nrLSU region. The primer sets 96F/938R (Uehling et al. 2012a) and RPB2-6F/fRPB2-7cR (Liu et al. 1999) were used to amplify the *rpb2* region. PCRs were conducted in a 25 μL reaction volume containing 2 μL of DNA template, 1 μL of each primer (10 μM), 8.5 μL of deionized water and 12.5 μL of 2 × Master Mix [Mei5 Biotechnology, Co., Ltd., China]. The PCR amplification conditions for nrITS and nrLSU refer to Li et al. (2020) and Sui et al. (2023). The PCR amplification conditions for *rpb2* refers to Salas-Lizana et al. (2023) and Huang et al. (2023). All the DNA sequences were sequenced by Sangon Biotech Co., Ltd. (Shanghai).

### ﻿Molecular phylogenetic analyses

The newly obtained sequences from this study were submitted to NCBI (https://www.ncbi.nlm.nih.gov). The nrITS, nrLSU, and *rpb2* sequences of the concatenated nrITS-nrLSU-*rpb2* datasets were aligned with selected sequences from GenBank and previous studies. All sequences are listed in Table 1. The generated raw reads of the DNA sequences were used to obtain consensus sequences using SeqMan v.7.1.0 in the DNASTAR Lasergene Core Suite software (DNASTAR Inc., Madison, WI, USA). All sequences were aligned using MAFFT v.6 (Katoh and Toh 2010) and trimmed manually with MEGA 6 (Tamura et al. 2013). For phylogenetic analyses, newly obtained sequences and additional reference sequences of *Clavulina* species were included in the dataset of the combined nrITS-nrLSU-*rpb2* fragment (Table 1), with *Hydnumrepandum* L. and *Hydnumrufescens* Pers. as the outgroups following He et al. (2016).

**Table 1.** Specimens used in phylogenetic analysis and their GenBank accession numbers. The newly generated sequences are shown in bold.

Taxonomy	Location	Voucher	GenBank Number		
			nrITS	nrLSU	*rpb2*
*Clavulinaalba*	Brazil	AMO869	ON502612	–	–
*C.alba*	Brazil	AMO868	ON502611	MZ484637	–
*C.alba*	Brazil	AMO867	ON502610	MZ484636	OQ305446
*C.alpina*	Italy	AMB n. 17156 (T)	MH456956	MH457104	–
*C.alpina*	Italy	AMB n. 17157	MH456957	MH457105	–
*C.amazonensis*	Guyana	TH8742	–	HQ680361	JN228249
*C.amazonensis*	Colombia	AMV1847	KT724111	KT724124	–
*C.amazonensis*	Brazil	AMO1143	ON502605	–	–
*C.amethystina*	Germany	MTH2	MN959776	–	–
*C.arboreiparva*	Mexico	FCME27282 (T)	NR_185406	MT903233	MK519629
*C.arboreiparva*	Mexico	MEXU 28239	MK547192	MT903234	–
*C.baiyunensis*	China	B21062720	OP738992	OP737358	–
*C.baiyunensis*	China	B21051526	OP738990	OP737357	OP745528
*C.baiyunensis*	China	B21062712 (T)	OP738991	OP737359	OP745529
*C.brunneocinerea*	New Zealand	TN42667	–	JN228220	–
*C.caespitosa*	Guyana	BRG:TH8709 (T)	NR_119560	DQ056370	JN228234
*C.castaneipes*	USA	OSC 116725	EU669210	EU669262	–
*C.castaneipes*	Costa Rica	TENN056432	JX287357	–	–
*C.castaneipes*	USA	OSC 108705	EU669209	EU669261	–
*C.cerebriformis*	Guyana	BRG:MCA4022 (T)	NR_121504	JN228222	JN228233
C.cf.amethystina	Norway	PRM 896664	EU862203	–	–
C.cf.amethystina	Norway	O 62152	EU862204	–	–
C.cf.amethystina	USA	SE-2015	KT275670	–	–
C.cf.cinerea	China	MES427	–	JN228226	JN228239
C.cf.cinerea	Canada	UBC:F29630	MZ868607	–	–
C.cf.cinerea	Canada	UBC:F29600	MZ868605	–	–
C.cf.cinerea	Spain	BIO 10304	EU862226	–	–
C.cf.cinerea	Spain	BIO 10294	EU862225	–	–
C.cf.connata	Guyana	TH 9586	JN247429	–	JN228247
C.cf.cristata	China	MES426	JN228225	JN228225	JN228240
C.cf.rugosa	China	MHHNU 9234	MK564142	MK564132	MK564152
***C.chengdeensis***	**China**	**BJTC TX646 (T)**	**PP835331**	**PP835344**	**PP889517**
***C.chengdeensis***	**China**	**BJTC 0249**	**PP835325**	**PP835339**	**PP889513**
***C.chengdeensis***	**China**	**BJTC ZH1205**	**PP835335**	**PP835348**	**PP889521**
***C.chengdeensis***	**China**	**BJTC ZH1225**	**PP835336**	**PP835349**	**PP889522**
*C.cinerea*	USA	iNat62500762	ON479751	–	–
*C.cinerea*	USA	ECV4030	MG663298	MF797670	–
*C.cinerea*	USA	JKU9	JN228228	–	JN228242
*C.cinerea*	Denmark	JV01-158	AJ889937	AJ889937	–
*C.cinerea*	Finland	KHL 11694 (GB)	EU118616	–	–
*C.cinerea*	USA	RAS494	OR471189	OR470998	OR474029
*C.cinereoglebosa*	Guyana	TH8561 (T)	NR_119975	JN228232	JN228246
*C.cirrhata*	Guyana	TH8940	JQ677059	JQ677045	J0677046
*C.cirrhata*	Guyana	TH8754	JQ677050	JQ677050	–
*C.cirrhata*	Guyana	TH9266	JQ677061	–	–
*C.cirrhata*	Guyana	TH9207	JQ677062	–	–
*C.coralloides*	USA	iNat61787378	ON479748	–	–
*C.coralloides*	–	HBAU15770	MW850398	–	–
*C.coralloides*	China	110116MFBPL0405	MW554410	–	–
*C.coralloides*	China	HKAS122411	ON794279	–	–
*C.craterelloides*	Colombia	AMV1401	–	KT724127	–
*C.craterelloides*	Guyana	TH8234 (T)	JQ911749	AY391718	–
*C.cristata*	USA	JKU8	JN228227	JN228227	JN228241
*C.cristata*	Spain	BIO 9641	EU862228	–	–
*C.cristata*	Spain	BIO 10291	EU862223	–	–
*C.cristata*	Finland	EL 6/00 (GB)	KF218965	KF218965	–
*C.cristata*	Sweden	GB/EL95-97	AY463398	AY586648	–
*C.cristata*	USA	DUKE9312	–	JN228215	JN228250
*C.cristata*	USA	RAS323 SV1	OR464379	OR460871	–
*C.cristata*	USA	RAS323 SV2	OR464380	OR460872	–
*C.crystallifera*	Brazil	AMO825	–	MZ484638	–
*C.crystallifera*	Brazil	URM 95027 (T)	–	MZ484639	OQ305455
*C.crystallifera*	Brazil	URM 95029	–	MZ484641	OQ305456
*C.cystidiata*	Brazil	URM 95030	NR_185725	MZ484642	OQ305449
*C.dicymbetorum*	Guyana	TH8730 (T)	DQ056364	DQ056369	–
*C.effusa*	Guyana	TH9193 (T)	–	JN228230	JN228245
*C.effusa*	Colombia	AMV1837	KT724116	KT724129	–
*C.effusa*	Guyana	TH8511	JN228231	–	–
*C.etruriae*	Italy	AMB n.17158	MH456958	–	–
*C.flava*	China	MHHNU 9811	MK564138	MK564128	MK564148
*C.flava*	China	MHHNU9825 (T)	NR_185562	MK564129	MK564149
*C.floridana*	Mexico	MEXU:28240	MK547187	MT903230	MK519625
*C.floridana*	Mexico	UNAM:FCME27277	MK547188	MT903229	MK519626
*C.floridana*	USA	Franck 4420 (T)	MT894294	MT894296	–
***C.griseoviolacea***	**China**	**BJTC ZH0998 (T)**	**PP835334**	**PP835347**	**PP889520**
***C.griseoviolacea***	**China**	**BJTC ZH1653**	**PP835338**	**PP835352**	**PP889524**
*C.grisea*	Brazil	URM 89966	KX811198	KX811193	–
*C.grisea*	Brazil	URM 89967 (T)	KX811199	KX811194	–
*C.grisea*	Brazil	URM 89968	KX811200	KX811195	–
*C.guyanensis*	Guyana	TH9257	JQ677057	–	–
*C.guyanensis*	Guyana	TH9245 (T)	NR_120085	–	JQ677049
*C.humicola*	Guyana	TH8737 (T)	DQ056368	DQ056367	JN228244
*C.incrustata*	Brazil	AMO1300	ON502606	MZ484631	OQ305439
*C.incrustata*	Brazil	AMO800B	–	MZ484626	OQ305440
*C.incrustata*	Brazil	AMO802	–	MZ484628	OQ305441
C.irisvar.iris	Cyprus	ML5135C1	MN028412	MN028396	–
C.irisvar.iris	China	QHU20388	OM970954	–	–
C.irisvar.occidentalis	France	PAM11112702	MN028408	–	–
C.irisvar.occidentalis	France	PAM12112740	MN028409	–	–
*C.junduensis*	Brazil	ANMF 766	MZ092866	–	–
*C.kunmudlutsa*	Guyana	MCA3117	–	HQ680362	–
*C.lilaceorosea*	China	B22052918	OP738996	OP737363	OP745532
*C.lilaceorosea*	China	B21082106	OP738995	OP737362	OP745533
*C.livida*	China	MCCNNU00959	KU219603	–	–
*C.livida*	China	MCCNNU00960 (T)	KU219605	KU219602	–
*C.mahiscolorata*	Mexico	FCME 27660	MH542551	MN049493	–
*C.mahiscolorata*	Mexico	FCME 27665	MH542553	–	–
*C.mahiscolorata*	Mexico	FCME 27662 (T)	MH542554	MN049496	MN053719
*C.minor*	China	B21081646 (T)	OP738993	OP737360	OP745530
*C.minor*	China	B22082717	OR149156	OR145333	OR166807
*C.monodiminutiva*	Guyana	TH8738 (T)	NR_119559	DQ056372	JN228237
*C.nigricans*	Guyana	TH8284 (T)	JN228224	AY391719	JN228238
*C.nigricans*	Guyana	G200	KJ786649	KJ786553	–
*C.ornatipes*	USA	iNAT:57799374	MW031157	–	–
*C.ornatipes*	USA	TH9598	–	JN228229	JN228243
*C.ossea*	Brazil	AMO1110	–	MZ484634	OQ305444
*C.ossea*	Brazil	AMO1108	ON502607	MZ484633	OQ305443
*C.pakaraimensis*	Guyana	TH9194 (T)	NR_121533	JQ677051	JQ677047
*C.pakaraimensis*	Guyana	TH9212	JQ677053	–	–
***C.pallida***	**China**	**BJTC C669 (T)**	**PP835329**	**PP835342**	**PP889515**
***C.pallida***	**China**	**BJTC C229**	**PP835327**	**PP835341**	**PP889514**
***C.pallida***	**China**	**BJTC S062**	**PP835330**	**PP835343**	**PP889516**
***C.pallida***	**China**	**BJTC ZH0056**	**PP835332**	**PP835345**	**PP889518**
***C.pallida***	**China**	**BJTC ZH0810**	**PP835333**	**PP835346**	**PP889519**
***C.pallida***	**China**	**BJTC ZH1055**	**PP835337**	**PP835350**	**PP889523**
*C.paraincrustata*	Brazil	URM 89969 (T)	–	KX811196	
*C.paraincrustata*	Brazil	AMO419	KX811201	–	–
*C.parvispora*	Mexico	FCME 27650 (T)	MH542550	MN049492	MN053718
*C.parvispora*	Mexico	FCME 27657	MH542549	MN049491	–
*C.perplexa*	Italy	AMB 19286	OR094916	OR095022	–
*C.perplexa*	Italy	AMB 19287	OR094915	OR095021	–
*C.purpurascens*	China	MHHNU 9846	MK564136	MK564126	MK564146
*C.purpurascens*	China	MHHNU 9848 (T)	MK564137	MK564127	MK564147
*C.reae*	Mexico	FCME 27623	MH542526	MN049487	MN053717
*C.reae*	Italy	AMB n. 17159	MH456959	MH457106	–
*C.reae*	Mexico	FCME 27629	MH542525	MN049488	–
*C.rosiramea*	Guyana	TH8954 (T)	JQ677064	JQ677044	JQ677048
*C.rugosa*	Tunisia	H21587	KU973837	–	–
*C.rugosa*	Spain	BIO 10293	EU862220	–	–
*C.rugosa*	Spain	BIO 10300	EU862217	–	–
*C.rugosa*	Spain	BIO 11162	EU862229	–	–
*C.rugosa*	Italy	AMB 19288	OR641046	OR641045	–
*C.rugosa*	USA	RAS487 SV1	OR464375	OR460869	OR473737
*C.rugosa*	USA	RAS327 SV2	OR464364	OR460870	–
*C.rugosa*	USA	RAS327 SV1	OR464365	–	OR473740
*C.samuelsii*	USA	TENN065723	JQ638712	–	–
*C.samuelsii*	New Zealand	PDD:89881	GU222317	–	–
*C.simplex*	Brazil	URM 95031 (T)	NR_185726	MZ484643	OQ305452
*C.simplex*	Brazil	URM 95032	–	MZ484644	–
*C.sphaeropedunculata*	Mexico	FCME 27661	MH542560	MK253716	–
*C.sphaeropedunculata*	Mexico	MEXU 28222 (T)	MH542557	MK253717	–
*C.sprucei*	Guyana	TH9122	HQ680355	JN228223	JN228236
*C.sprucei*	Guyana	TH9120	HQ680353	–	–
*C.sprucei*	Guyana	MCA3989	HQ680352	–	JN228235
*C.studerae*	Brazil	URM 95036	–	MZ484648	OQ305454
*C.studerae*	Brazil	URM 95033 (T)	NR_185727	MZ484645	OQ305453
*C.subrugosa*	USA	TENN043395	JQ638711	–	–
*C.subrugosa*	New Zealand	TN43395	JN228221	–	–
*C.tepurumenga*	Guyana	MCA3116	–	HQ680363	JN228248
*C.terminalis*	Brazil	AMO1109	–	MZ484649	OQ305450
*C.terminalis*	Brazil	AMO1122	–	MZ484651	OQ305451
*C.thindii*	China	dcy2288	MZ157027	–	–
*C.thindii*	India	US_1428	MG892054	–	–
*C.tropica*	China	ZP3327	ON898016	–	–
*C.tropica*	China	MHHNU 9827	ON954848	–	–
*C.tuxtlasana*	Mexico	MEXU 28242	MK547194	MT903236	MK519631
*C.tuxtlasana*	Mexico	MEXU 28243	MK547195	MT903237	MK519632
*C.tuxtlasana*	Mexico	UNAM:FCME27279 (T)	NR_185407	MT903238	MK519633
*Hydnumrepandum*	Slovenia	031209A	KU612574	KU612655	–
*H.rufescens*	Slovenia	LJU GIS 1332	AJ547868	–	–
*H.rufescens*	China	HKAS82529	–	KU612657	–

To estimate Maximum Likelihood (ML) phylogenetic trees, we utilized RAxML 7.4.2 Black Box software (Stamatakis 2006; Stamatakis et al. 2008; Zhou and Hou 2019; Zhou et al. 2021) with a GTRGAMMAI site substitution model (Guindon et al. 2010). Branch support was calculated with a bootstrapping (BS) method of 1000 replicates (Hillis and Bull 1993). Bayesian Inference (BI) analysis was conducted using MrBayes 3.1.2 (Ronquist and Huelsenbeck 2003) with a Markov chain Monte Carlo (MCMC) algorithm (Rannala and Yang 1996). The best model was estimated using MrModeltest 2.3 (Zhou and Hou 2019; Zhou et al. 2021, 2022). The models employed for each marker of the nrITS-nrLSU-*rpb2* dataset were HKY + G for nrITS, GTR + I + G for nrLSU, and HKY + I + G for *rpb2*. We ran two MCMC chains for 100,000,000 generations, stopping when the average standard deviation of split frequencies dropped below 0.01. Trees were saved every 1000 generations, and the initial 25% of trees were discarded as the burn-in phase for each analysis. Significant Bayesian posterior probabilities were calculated for branches in the remaining trees, as the analysis yielded relatively stable topologies, and clades with high Bayesian posterior probability (pp) values reflected the relative relationships between species (Posada and Crandall 1998).

## ﻿Results

### ﻿Molecular phylogeny

**Figure 1.**
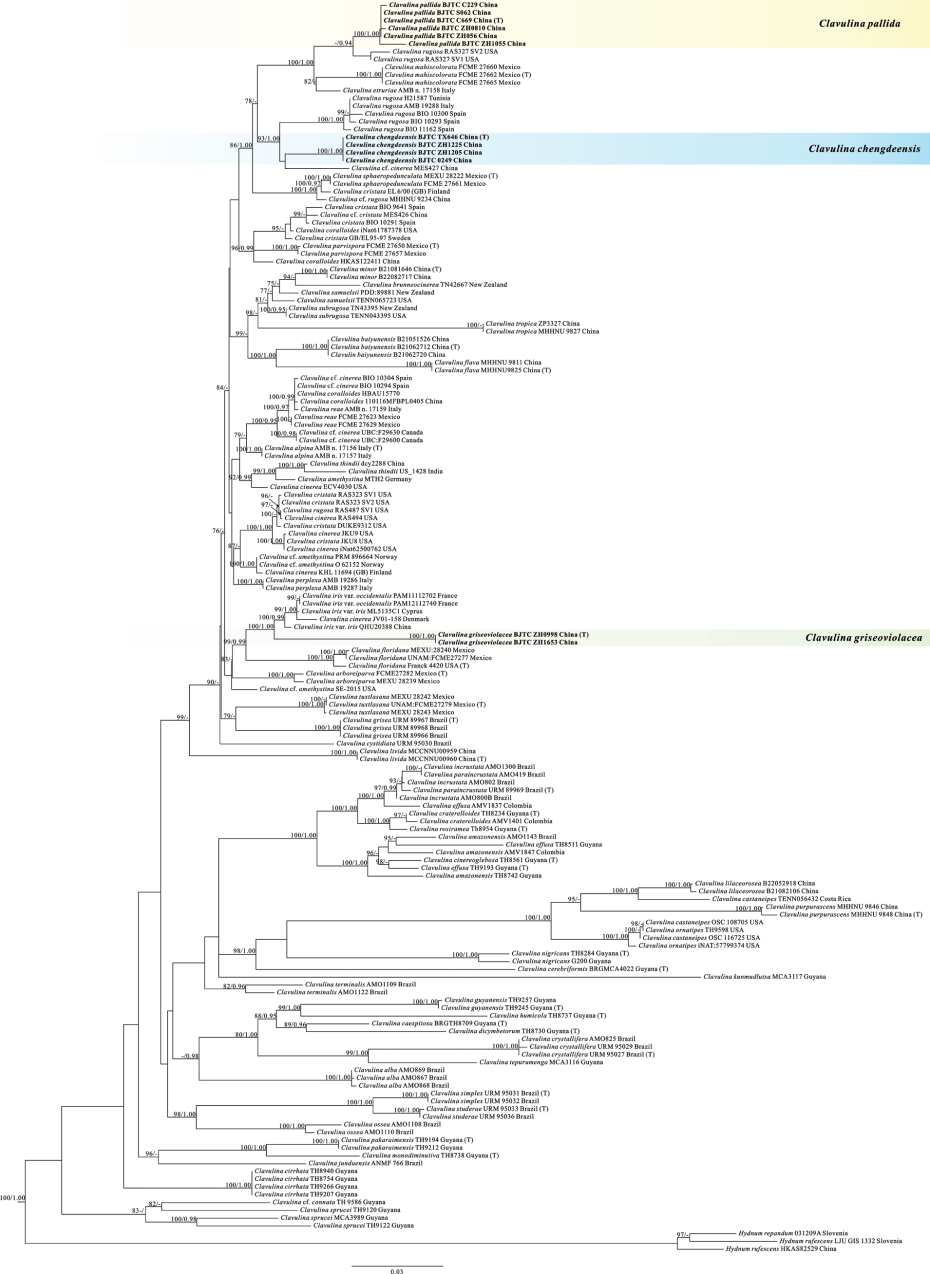
Phylogenetic tree generated from a ML analysis based on combined nrITS-nrLSU-*rpb2* sequences. Numbers representing Maximum Likelihood bootstrap support (MLBS ≥ 75%, right) and significant Bayesian posterior probability (BPP ≥ 0.95, left) are indicated above the nodes. Novel sequences are printed in bold. Voucher specimens and localities where the specimens were collected are provided behind the species names.

A total of 36 sequences, including 12 for nrITS, 12 for nrLSU and 12 for *rpb2*, were generated in this study. The nrITS-nrLSU-*rpb2* dataset included 320 sequences (140 for nrITS, 109 for nrLSU, and 71 for *rpb2*), with 161 samples. The concatenated alignment contained 2188 characters, including gaps. ML and Bayesian analyses resulted in highly similar estimates of tree topologies; thus, only the tree inferred from the ML analysis is shown (Fig. 1).

Our tree topology is similar to that of de Meiras-Ottoni and Gibertoni (2023). On the basis of our analyses, three highly supported monophyletic lineages were identified in our *Clavulina* samples (Fig. 1). Morphological examinations revealed that these species are morphologically different from other known species of this genus. The three clades therefore can be recognized as three new species and described in this paper, i.e., *Clavulinachengdeensis* sp. nov., *Clavulinagriseoviolacea* sp. nov., and *Clavulinapallida* sp. nov. Moreover, *Clavulinagriseoviolacea* (BJTC ZH0998 and BJTC ZH1653) further clustered a clade with Clavulinairisvar.iris Loizides, Bellanger & P.-A. Moreau, Clavulinairisvar.occidentalis Bellanger, P.-A. Moreau & Loizides, and *Clavulinacinerea* (MLB = 100%, BPP = 1.00). *Clavulinapallida* (BJTC C669, BJTC C229, BJTC S062, BJTC ZH0056, BJTC ZH0810, and BJTC ZH1055) formed one clade (MLB = 100%, BPP = 1.00) closely related to *Clavulinarugosa*, *Clavulinamahiscolorata* E. Pérez-Pazos & Villegas, and *Clavulinaetruriae* Franchi & M. Marchetti (MLB = 100%, BPP = 1.00) (Fig. 1). The sequences of *Clavulinachengdeensis* (BJTC TX646, BJTC 0249, BJTC ZH1205, and BJTC 1225) formed a branch with a high support value (MLB = 100%, BPP = 1.00) and further clustered into a clade with *Clavulinarugosa* and Clavulinacf.cinerea (Bull.) J. Schröt.), which was well supported (MLB = 93%, BPP = 1.00).

### ﻿Taxonomy

#### ﻿ Clavulina chengdeensis

Taxon classificationFungiCantharellalesHydnaceae

Yue Gao, Hao Zhou, & C.L. Hou sp. nov.

784F58C2-DD2D-5B07-90EE-98D0F70E15D6

MycoBank No: 853047

**Figure 2.**
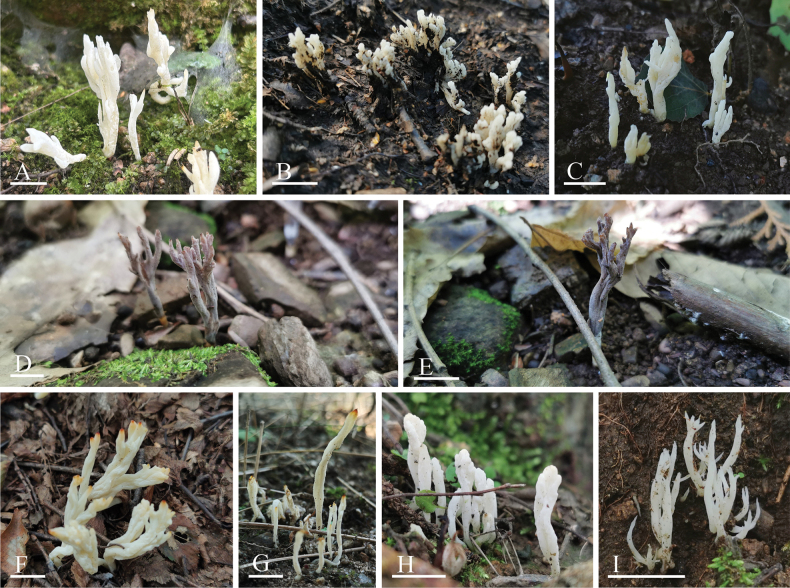
Morphology of Basidiomata **A–C***Clavulinachengdeensis* (**A**BJTC TX646 holotype **B**BJTC ZH1205 **C**BJTC ZH1225) **D–E***Clavulinagriseoviolacea* (**D**BJTC ZH0998 holotype **E**BJTC ZH1653) **F–I***Clavulinapallida* (**F**BJTC C669 holotype **G**BJTC S062 **H**BJTC ZH1055 **I**BJTC C229). Scale bars: 1 cm.

**Figure 3.**
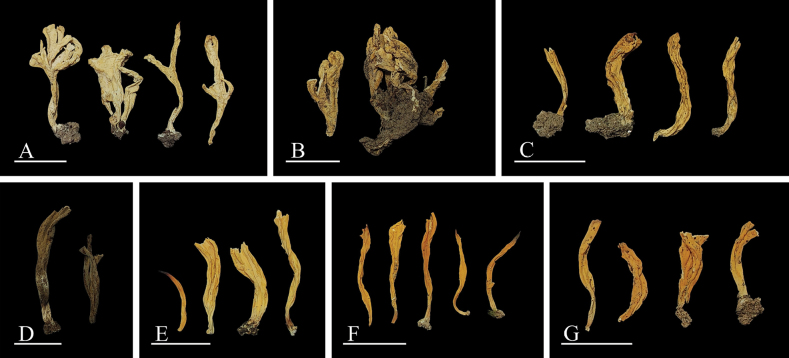
Morphology of Basidiomata **A–C***Clavulinachengdeensis* (**A**BJTC TX646 holotype **B**BJTC ZH1205 **C**BJTC 0249) **D***Clavulinagriseoviolacea* (**D**BJTC ZH0998 holotype) **E–G***Clavulinapallida* (**E**BJTC C669 holotype **F**BJTC S062 **G**BJTC ZH0056). Scale bars: 1 cm.

**Figure 4.**
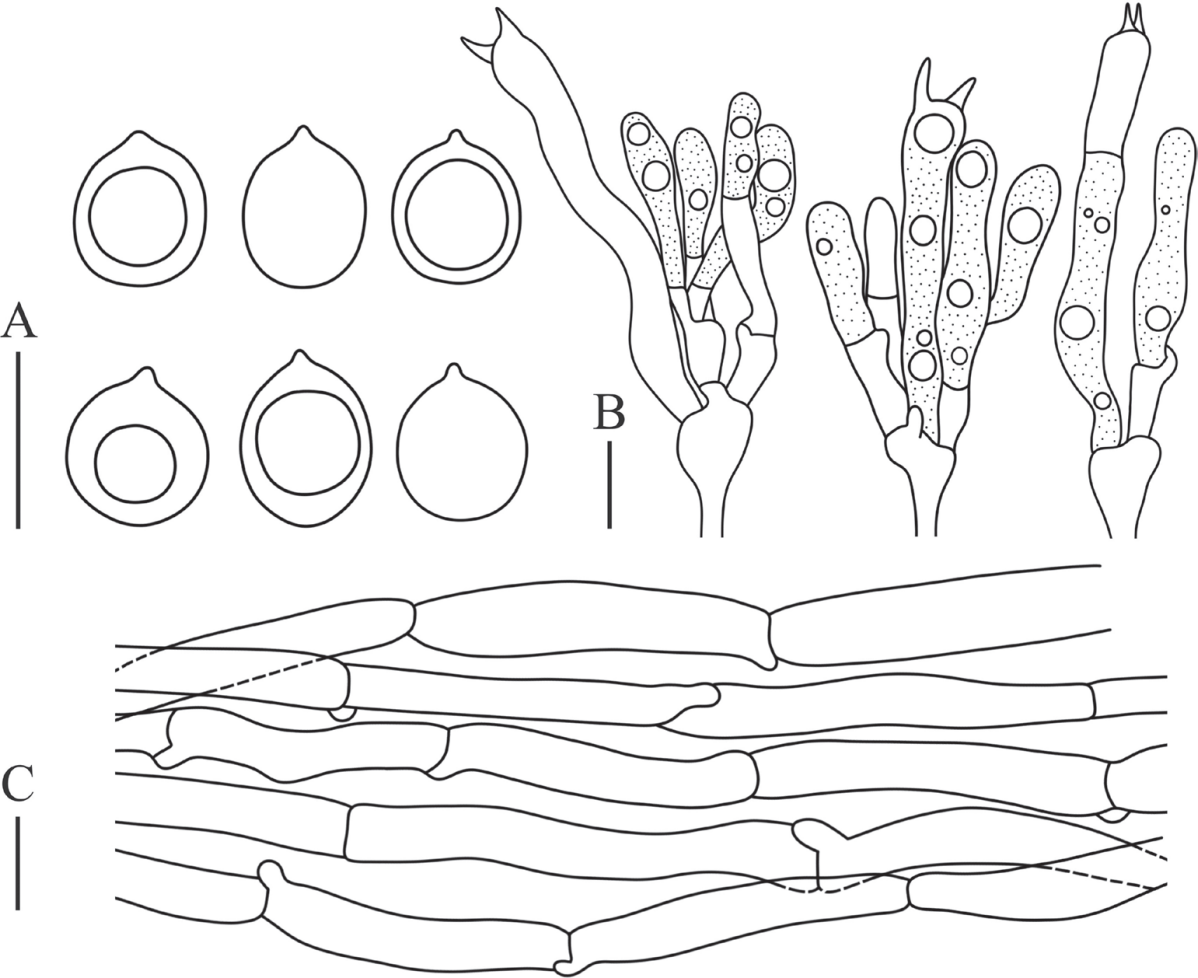
Microscopic characteristics of *Clavulinachengdeensis***A** basidiospores **B** basidia **C** tramal hyphae. Scale bars: 10 µm.

Figs 2A–C , 3A–C , 4

##### Diagnosis.

*Clavulinachengdeensis* differs from known *Clavulina* species in its white to dirty white basidiomata with somewhat pale orange tips, somewhat wrinkled hymenium, basidiospores 6.1–9.6 × 5.6–7.9 μm, basidia 40.1–58.7 × 4.5–7.0 μm, postpartal septa present, and clamp connections present.

##### Etymology.

The epithet “*chengdeensis*” refers to the specimens collected from Chengde city.

##### Type.

China • Hebei Province, Chengde city, Xiaolabagou; 40°57'24"N, 116°27'12"E, alt. 1240 m; 13 Sep. 2023; H. Zhou, Y. Gao & X. Tong (BJTC TX646); GenBank nrITS: PP835331, nrLSU: PP835344, *rpb2*: PP889517.

##### Description.

Basidiomata coralloid, solitary or gregarious in cespitose clusters; clusters 12–24 mm tall, 10–26 mm wide across branches and forming 3–5 ranks in multiple planes; individual basidiomata 14–40 mm tall, 4–11 mm wide, simple or sparsely branched one time, branching pattern polychotomous to dichotomous ascending, branches subterete or subclavate and somewhat flattened with age, rough, branch tips rounded when young, gradually become pointed with age; white (#ffffff) to dirty white (#fff9eb) when fresh and somewhat pale orange (#ffeab8), cream white (#fff2d2) to light grayish orange (#f6e4d0) when dry. Stipes generally distinct, 5–15 mm long, 1.5–4 mm wide, subcylindrical or flattened, concolor with branches. Hymenium amphigenous, somewhat wrinkled.

Basidiospores [87/9/4] (5.7−) 6.1–9.6 (−11.0) × 5.6–7.9 (−8.5) μm, *Q* = 1.00–1.25 (−1.31), *Q_m_* = 1.04 ± 0.02, globose to subglobose, smooth, hyaline in H_2_O and KOH, thin-walled, inamyloid, with a 0.6–1.0 μm irregular hilar appendix, and one large oleiferous guttule. Basidia (34.7–) 40.1–58.7 (−62.3) × 4.5–7.0 (−8.3) μm, clavate to subcylindrical, tapering from apex to base; postpartal septa present in most basidia, occurring 11–30 μm below basidia tips; two sterigmata occur per basidium, 5.1–7.5 μm long, and cornute. Basidioles abundant, subclavate to subcylindrical. Tramal hyphae in stipe smooth, with slightly thickened walls, hyaline in KOH, 4.0–7.7 μm wide, sometimes inflated to 9.3 μm wide; tramal hyphae in branches hyaline, thin-walled, sometimes inflated tramal hyphae 3.7–8.5 (−10.4) μm wide; clamp connections abundant. Hyphal system monomitic. Cystidia absent.

##### Habit, habitat, and distribution.

Solitary or gregarious caespitose in humus layers on soils in broad-leaved deciduous forests associated with *Castanea* Mill., *Betula* L., and *Platanus* L. Basidiomata generally occur from July to September; currently known from Hebei Province and Beijing, China.

##### Additional specimens examined.

China • Beijing, Yanqing District, Songshan National Nature Reserve; 40°30'N, 115°48'E, alt. 652 m; 16 Sep. 2017; C.L. Hou, H. Zhou, J.Q. Li (BJTC 0249); China• Beijing, Huairou District, Labagoumen Na­ture Reserve; 40°39'24"N, 116°27'14"E, alt. 1225 m; 25 Aug. 2021; H. Zhou, X.Y. Shen, X.B. Huang (BJTC ZH1205); same location• 40°57'17"N, 116°27'3"E, alt. 1308 m; 25 Aug. 2021; H. Zhou, X.Y. Shen, X.B. Huang (BJTC ZH1225).

##### Note.

Phylogenetically, *C.chengdeensis* was related to *C.rugosa* in our analyses (Fig. 1). However, *C.rugosa* has longitudinally rugulose basidiomata, relatively large basidiospores (9–14 × 8–12 µm), basidia (40–85 × 6.9–5 µm), and sterigmata 6–9 µm long (Corner 1950; Olariaga et al. 2009). Morphologically, *C.chengdeensis* is close to *C.mahiscolorata*. *Clavulinamahiscolorata* generally shares similar colors with basidiomata, but its basidiomata turn maize yellow to mandarin orange when dry and the branch surface is smooth (Pérez-Pazos et al. 2019).

#### ﻿ Clavulina griseoviolacea

Taxon classificationFungiCantharellalesHydnaceae

Yue Gao, Hao Zhou, & C.L. Hou sp. nov.

713DBA60-9517-57D2-8850-C1A8A29BA820

MycoBank No: 853050

Figs 2D–E , 3D , 5

##### Diagnosis.

*Clavulinagriseoviolacea* differs from known *Clavulina* species in its gray to dark grayish violet basidiomata, with a white stipe, monopodial or irregularly polychotomous branches toward branch apices, basidiospores 6.5–8.0 × 6.2–7.2 μm, basidia 31.3–49.8 × 4.5–7.2 μm, postpartal septa present, and clamp connections present.

##### Etymology.

The epithet “*griseoviolacea*” refers to the basidiomata being gray to dark grayish violet.

##### Type.

China • Tianjin, Jizhou District, Jiulongshan; 40°8'51"N, 117°30'36"E, alt. 170 m; 21 Aug. 2022; H. Zhou, X.Y. Shen & X.B. Huang (BJTC ZH0998); Gen­Bank nrITS: PP835334, nrLSU: PP835347, *rpb2*: PP889520.

**Figure 5.**
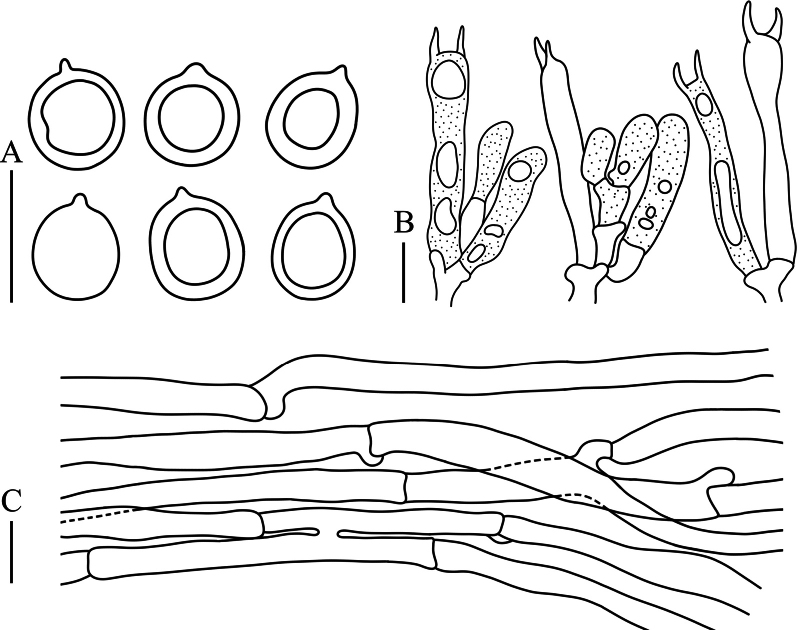
Microscopic characteristics of *Clavulinagriseoviolacea***A** basidiospores **B** basidia **C** tramal hyphae. Scale bars: 10 µm.

##### Description.

Basidiomata coralloid, solitary or scattered; individual basidiomata 25–45 mm tall, 12–20 mm wide across branches, basidiomata sparsely branched two to three times, monopodial or irregularly polychotomous toward branch apices and dichotomous at the base, branches subclavated to fattened and somewhat flattened with age, rough, with rounded tips; gray (#a5a5a5) to dark grayish violet (#9b92a6) when fresh and tips somewhat very dark brown (#1f1605), dark brown (#4c350b) when dry. Stipes generally distinct, 10–20 mm long, 2–5 mm wide, subcylindrical or flattened, dark gray (#777777) to dark grayish purple (#7a747f) and sometimes with a white base (#ffffff). Hymenium amphigenous, generally nodulosus and farinaceous.

Basidiospores [65/2/1] 6.5–8.0 (−8.6) × (5.9−) 6.2–7.2 (−7.9) μm, *Q* = 1.00–1.23 (−1.27), *Q_m_* = 1.12 ± 0.06, globose to subglobose, smooth, hyaline in H_2_O and KOH, thin-walled, inamyloid, with a 0.6–0.9 μm irregular hilar appendix, and one large oleiferous guttule. Basidia (29.3–) 31.3–49.8 (−65.3) × 4.5–7.2 (−8.3) μm, clavate to subcylindrical, tapering from apex to base; postpartal septa present in most basidia, occurring 12–24 μm below basidia tips; two sterigmata occur per basidium, 3.8–6.7 μm long, and cornute. Basidioles abundant, subclavate to subcylindrical. Tramal hyphae in stipe smooth, with slightly thickened walls, hyaline in KOH, 2.6–5.5 μm wide, some tramal hyphae inflated; tramal hyphae in branch hyaline, thin-walled, 3.7–6.1 (−8.9) μm wide; clamp connections abundant. Hyphal system monomitic. Cystidia absent.

##### Habit, habitat, and distribution.

Solitary or scattered humus layers on soils under Theropencedrymion, associated with *Pinus* L. Basidiomata generally occurring from July to August; currently known from Tianjin and Beijing, China.

##### Additional specimens examined.

China • Beijing, Mentougou District, Baihua Mountain; 39°47'50"N, 115°33'35"E, alt. 1,223 m; 16 Aug. 2023; H. Zhou, Y. Gao & X. Tong (BJTC ZH1653).

##### Notes.

*C.griseoviolacea* is phylogenetically closely related to *Clavulinacinerea*, Clavulinairisvar.occidentalis and Clavulinairisvar.iris according to phylogenetic analyses (Fig. 1.), but *Clavulinacinerea* has lilac-gray to gray basidiomata, larger basidiospores (7–10 × 6–8 µm) and basidia (38–65 µm long up to 7 µm wide) (Burt 1922); *Clavulinairis* Loizides, Bellanger & P.-A. Moreau can be distinguished by its white-pruinose basidiomata, branches sometimes partially or extensively fused, larger basidiospores (9.2–10.4 × 6.5–8.5 µm) and basidia (45–80 × 6–9 µm), and 7–9 µm wide hyphal ends (pseudocystidia) (Crous et al. 2019). *C.griseoviolacea* is morphologically similar to *Clavulinatuxtlasana* M. Villegas, Garibay-Orijel & Pérez-Pazos in the color of basidiomata, but *C.tuxtlasana* basidiomata are simple, rarely branching dichotomously, branches with slight longitudinal wrinkles, smaller basidiospores (6–7.5 × 5.5–7 µm), and lacking clamp connections (Salas-Lizana et al. 2023). *Clavulinacrystallifera* Meiras-Ottoni also has similar branching, but acerose crystals are present in the medullary hyphae of its branches (de Meiras-Ottoni and Gibertoni 2023).

#### ﻿ Clavulina pallida

Taxon classificationFungiCantharellalesHydnaceae

Yue Gao, Hao Zhou, & C.L. Hou sp. nov.

89CBC465-3184-512C-A16B-7EBCA2F462B8

MycoBank No: 853052

**Figure 6.**
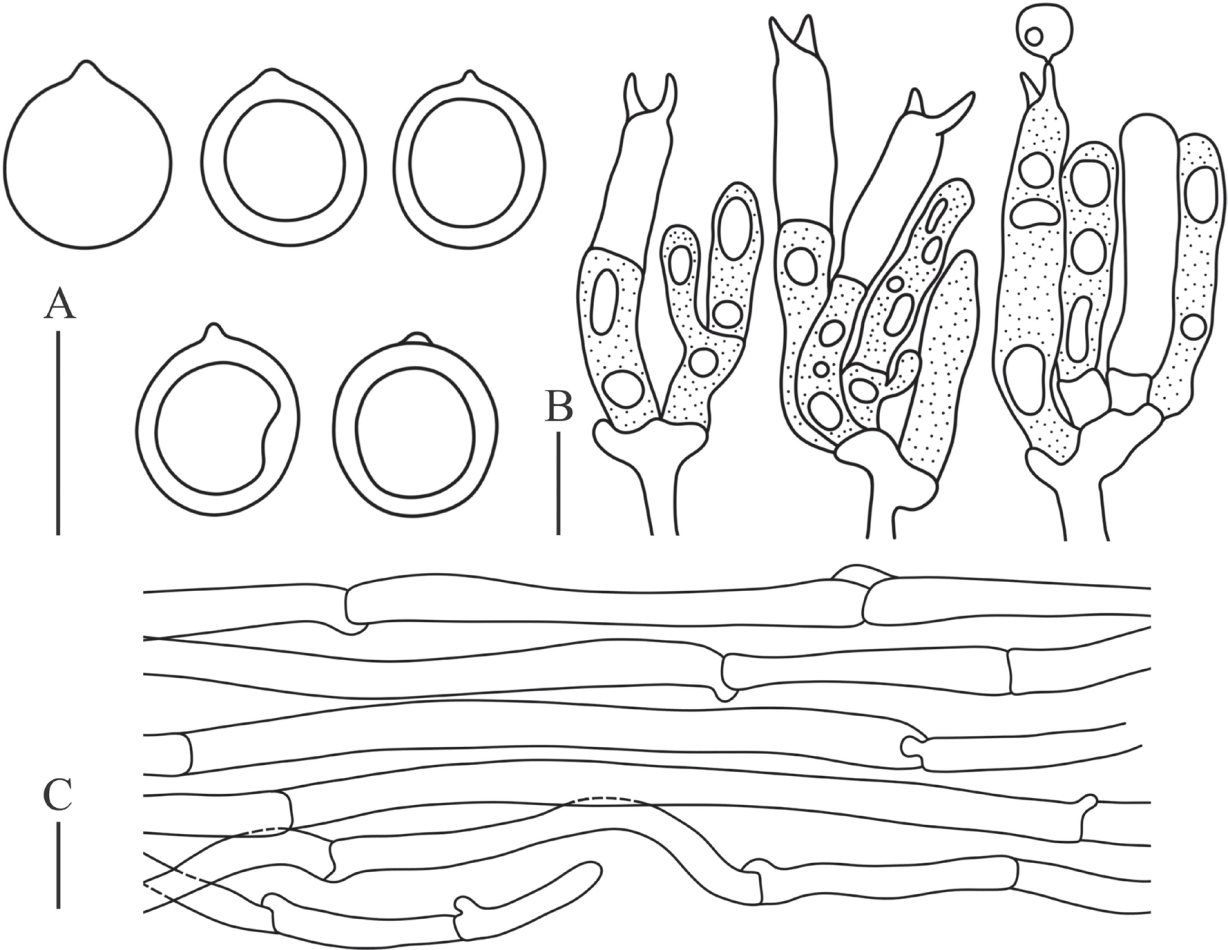
Microscopic characteristics of *Clavulinapallida***A** basidiospores **B** basidia **C** tramal hyphae. Scale bars: 10 µm.

Figs 2F–I , 3E–G , 6

##### Diagnosis.

*Clavulinapallida* differs from known *Clavulina* species in its white to pale cream white basidiomata, with somewhat orange tips, basidiospores 7.0–9.7 × 6.4–8.6 μm, basidia 34.2–48.5 × 4.8–6.3 μm, postpartal septa present, and clamp connections present.

##### Etymology.

The epithet “pallida” refers to the basidiomata being pale white.

##### Type.

China • Beijing, Miyun District, Heilongtan; 40°35'30"N, 116°46'20"E, alt. 397 m; 27 Aug. 2021; C.L. Hou, G.Q. Cheng, R.T. Zhang (BJTC C669); GenBank nrITS: PP835329, nrLSU: PP835342, *rpb2*: PP889515.

##### Description.

Basidiomata coralloid, solitary or scattered; individual basidiomata 17–38 mm tall, 1.8–10 mm wide across branches, simple or sparsely branched one to two times, monopodial or irregularly dichotomous toward branch apices and monopodial or dichotomous at the base, branches subterete or subclavate to fattened and somewhat flattened with age, smooth or rough, branch tips are rounded when young, gradually become pointed with age; white (#ffffff) to pale cream white (#fffaef) when fresh and tips somewhat orange (#ff9000), brownish orange (#cd8400) when dry. Stipes generally distinct, 7–14 mm long, 1.5–4 mm wide, subcylindrical or flattened, white (#ffffff) to pale cream white (#fffaef). Hymenium amphigenous, somewhat wrinkled.

Basidiospores [131/10/6] (6.6−) 7.0–9.7 (−10.1) × (6.0−) 6.4–8.6 (−10.1) μm, *Q* = 1.00–1.24 (−1.29), *Q_m_* = 1.10 ± 0.06, globose to subglobose, smooth, hyaline in H_2_O and KOH, thin-walled, inamyloid, with a 0.6–1.1 μm irregular hilar appendix, and one large oleiferous guttule. Basidia (31.8–) 34.2–48.5 (−55.3) × 4.8–6.3 (−7.6) μm, clavate to subcylindrical, tapering from apex to base; postpartal septa present in most basidia, which occurred 10–22 μm below basidia tips; two sterigmata occur per basidium, 3.7–6.2 μm long, and cornute. Basidioles abundant, subclavate to subcylindrical. Tramal hyphae in stipe smooth, thin walled, hyaline in KOH, 3.3–6.2 μm wide, not inflated; tramal hyphae in branch hyaline and thin-walled, 2.8–6.7 (−9.0) μm wide; clamp connections abundant. Hyphal system monomitic. Cystidia absent.

##### Habit, habitat, and distribution.

Solitary or scattered in humus layers on soils in broad-leaved deciduous forests associated with *Carpinus* L. and *Castanea* Mill. Basidiomata generally occur from July to August and are currently known from Beijing and Hebei Province, China.

##### Additional specimens examined.

China • Beijing, Pinggu District, Zhenluo Mountain Villa; 40°20'24"N, 117°8'57"E, alt. 294 m; 20 Aug. 2021; C.L. Hou, G.Q. Cheng, R.T. Zhang (BJTC C229); China • Beijing, Yanqing District, Heibei village; 40°25'48"N, 116°14'44"E, alt. 456 m; 11 Aug. 2018; X.Y. Shen, B.D. He, K.B. Huang (BJTC S062); China • Beijing, Changping District, Yanshou Tem­ple; 40°22'24"N, 116°19'22"E, alt. 276 m; 14 Aug. 2019; C.L. Hou, H. Zhou, G.Q. Cheng (BJTC ZH0056); same location • 40°22'6"N, 116°19'19"E, alt. 215 m; 17 Aug. 2021; H. Zhou, X.Y. Shen, X.B. Huang (BJTC ZH0810); China • Hebei Prov­ince, Zunhua City, Wanfo Garden; 40°11'34"N, 116°37'1"E, alt. 144 m; 22 Aug. 2021; H. Zhou, X.Y. Shen, X.B. Huang (BJTC ZH1055).

##### Notes.

In the phylogenetic analyses (Fig. 1.), *C.pallida* is phylogenetically close to *Clavulinamahiscolorata*. *C.mahiscolorata* also has white basidiomata, but turns orange when dried, larger basidia (56–74 × 6–8 µm) are present (Pérez-Pazos et al. 2019). As described in the current record of the genus *Clavulina*, in terms of macroscopic features, *Clavulinarugosa* is closely related to *C.pallida*, with simple or sparsely branched basidiomata. However, *C.rugosa* differs from *C.pallida* in that it has longitudinally wrinkled surfaces of basidiomata and relatively large basidiospores (9–14 × 8–12 µm) (Corner 1950; Olariaga et al. 2009). *Clavulinalivida* generally shares similar simple basidiomata with *C.pallida*, but the former has grayish olive to dark grayish olive basidiomata, branch tips becoming pale pinkish cinnamon to chestnut with age, larger basidiospores (11.6–12.9 × 10.7–12.5 µm) and basidia (51.5–76.7 × 7.0–12.3 µm) (He et al. 2016). *Clavulinacaespitosa* T.W. Henkel, Meszaros & Aime differs from *C.pallida* in its simple basidomata in cespitose clusters, free or slightly fused basally, apex sharply acuminate, larger basidiospores (8.5–10.5 × 7–9.5 µm) and basidia (81–98 µm long, apex 6.3–7.5 µm, at base 3.5–5 µm), and postpartal septa not observed (Henkel 2005).

## ﻿Discussion

In early studies on *Clavulina*, only brief records of the macroscopic and microscopic features of the species were made. This resulted in difficulties in distinguishing between similar genera or species and led to the occurrence of many synonymous names (Hibbett et al. 1997; Binder et al. 2005; Moncalvo et al. 2006; Larsson 2007; Uehling et al. 2012b). For example, *Clavulinaparaincrustata* Meiras-Ottoni & Gibertoni, described only by morphology from the Brazilian Atlantic rainforest (Tibpromma et al. 2017), is treated as a synonym of *C.incrustata*. Wartchow found the same region (Wartchow 2012) on the basis of molecular evidence (de Meiras-Ottoni and Gibertoni 2023).

Previous studies have indicated that Clavarioid fungi, including *Clavulina*, are highly diverse and distributed worldwide (Corner 1950). However, to date, only 11 species of *Clavulina* have been described in China. China has a vast territory, complex climate, habitat types, and abundant species resources, with extremely high fungal diversity (Mcneely et al. 1990). The lack of records in this case may be due to the limited number of collections and collection areas. In this study, three new species of *Clavulina* were described on the basis of samples collected from North China, by means of both nrITS-nrLSU-*rpb2* three-locus phylogenetic analyses (Fig. 1) and macrofungal morphological examinations, which increase understanding of the species diversity of this genus. The natural growth of *Clavulina* may be related to precipitation. However, the investigations and specimen collection in this study were carried out in the rainy season from August to September, with no collection in other periods. Therefore, more *Clavulina* species were likely to be present in the study area.

## Supplementary Material

XML Treatment for Clavulina chengdeensis

XML Treatment for Clavulina griseoviolacea

XML Treatment for Clavulina pallida

## ﻿Additional information

### Author ORCIDs

Yue Gao https://orcid.org/0009-0001-0831-3656

Xin Tong https://orcid.org/0000-0002-2091-6636

Hao Zhou https://orcid.org/0000-0002-4869-2187

Hai-Qi Wang https://orcid.org/0009-0004-3388-6263

Cheng Li https://orcid.org/0009-0005-0958-0456

Cheng-Lin Hou https://orcid.org/0000-0001-8162-5560

### Data availability

All of the data that support the findings of this study are available in the main text.
